# Supplementary material for: Distributed quantum sensing of multiple phases with fewer photons
Source: Nat Commun. 2024 Jan 11;15:266. doi: 10.1038/s41467-023-44204-z (PMC10784500; doi:10.1038/s41467-023-44204-z)
Supplement: Supplementary file 1 — Supplementary Information [file 41467_2023_44204_MOESM1_ESM.pdf]

Supplementary Information - Distributed quantum sensing of multiple phases with fewer photons

Dong-Hyun Kim,[1,2,*] Seongjin Hong,[3,*] Yong-Su Kim,[1,4] Yosep Kim,[1,5] Seung-Woo Lee,[1] Raphael C. Pooser,[6] Kyunghwan Oh,[2] Su-Yong Lee,[7,8] Changhyoup Lee,[9] and Hyang-Tag Lim[1,4,†]

[1]*Center for Quantum Information, Korea Institute of Science and Technology (KIST), Seoul, 02792, Korea*
[2]*Department of Physics, Yonsei University, Seoul 03722, Korea*
[3]*Department of Physics, Chung-Ang University, Seoul 06974, Korea*
[4]*Division of Nanoscience and Technology, KIST School, Korea University of Science and Technology, Seoul 02792, Korea*
[5]*Department of Physics, Korea University, Seoul 02841, Korea*
[6]*Oak Ridge National Laboratory, Oak Ridge, TN 37831, USA*
[7]*Emerging Science and Technology Directorate, Agency for Defense Development, Daejeon 34186, Korea*
[8]*Weapon Systems Engineering, ADD School, University of Science and Technology, Daejeon, 34060, Korea*
[9]*Korea Research Institute of Standards and Science, Daejeon 34113, Korea*

* These authors contributed equally
† hyangtag.lim@kist.re.kr

SUPPLEMENTARY NOTE 1 - EXPERIMENTAL DETAILS

A. Experimental method

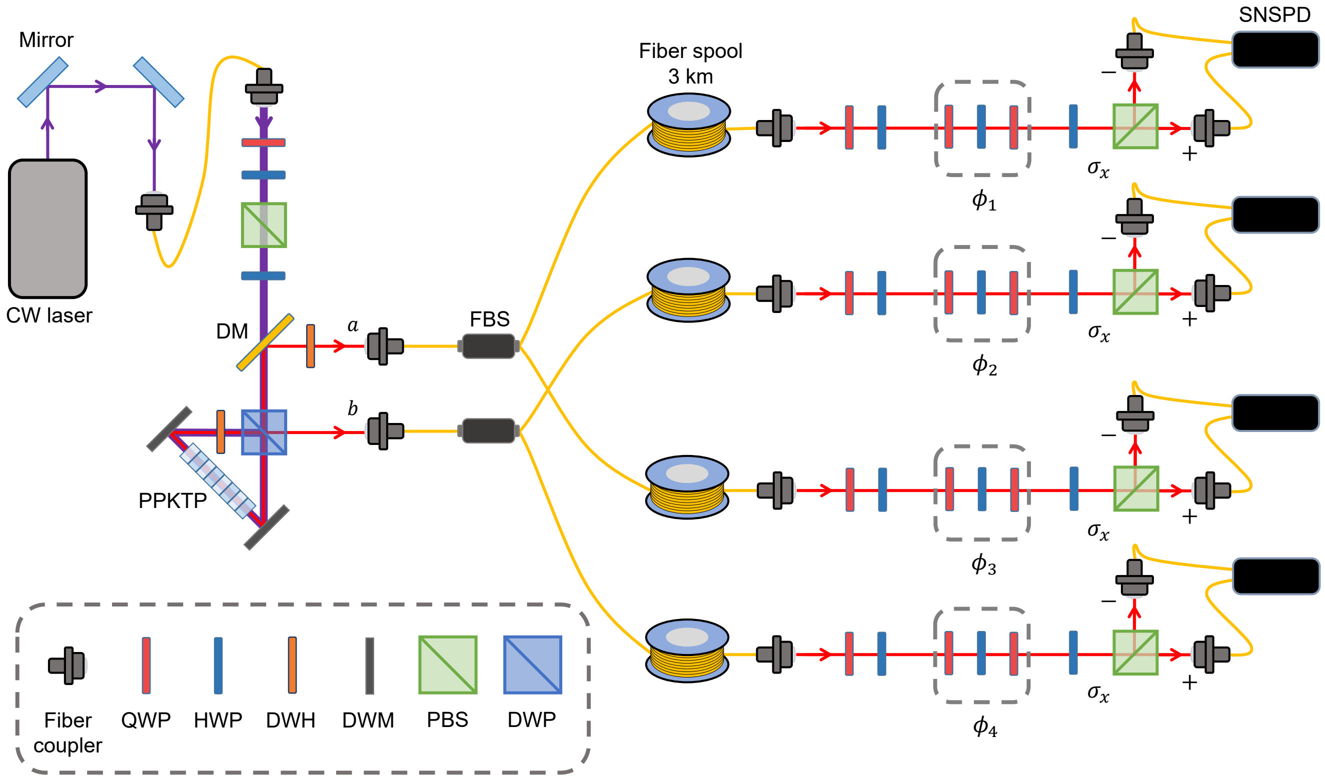

Supplementary Figure 1. **Experimental setup for distributed quantum sensing.** QWP: quarter waveplate; HWP: half waveplate; DWH: dual wavelength HWP; DWM: dual wavelength mirror; PBS: polarizing beam splitter; DWP: dual wavelength PBS; DM: dichroic mirror; PPKTP: periodical poled KTiOPO; FBS: 50/50 fiber beam splitter, SNSPD: superconducting nanowire single-photon detector.

Supplementary Figure 1 shows an experimental setup for demonstrating our distributed quantum sensing scenario, and corresponds to Figure 2 of the main text. We used a CW laser with a central wavelength of 780 nm as a pump laser, and the pump laser was sent to the Sagnac interferometer through a single mode fiber (SMF) for a spatial mode filtering. The polarization of the pump laser is set to $|H\rangle$ using a set of a quarter-wave plate (QWP), a half wave-plate (HWP), and a polarizing beam splitter (PBS). Then, the $|H\rangle$ pump laser is set to $|D\rangle{=}(|H\rangle{+}|V\rangle)/\sqrt{2}$ using a HWP with an optic axis angle of 22.5°. After passing through the dual wavelength PBS (DWP) working for both 780 nm and 1560 nm photons, the diagonal polarization of the pump laser is divided by horizontal and vertical polarizations, and the pump laser with $|H\rangle$ ($|V\rangle$) polarization goes through clockwise (counter-clockwise) direction inside a Sagnac interferometer. Note that $|V\rangle$ polarization part of the pump laser is set to the $|H\rangle$ polarization after transmitting a dual wavelength HWP (DWH), which works as a HWP for both 780 nm and 1560 nm photons. Then, the both of the counter-propagating pump beams probabilistically generate a pair of 1560 nm photons with orthogonal polarizations of $|H\rangle$ and $|V\rangle$ at the 10 mm-thick type-II periodically poled KTiOPO_4 (PPKTP) crystal with 46.2 µm polling period via spontaneous parametric down conversion (SPDC) process. After passing through a DWP, we can prepare the polarization Bell state $|H\rangle|V\rangle + e^{i\varphi}|V\rangle|H\rangle$ of 1560 nm, where φ denote a relative phase [1]. Then, using a DWH, we can prepare $|\Phi_{a,b}\rangle = (|H_aH_b\rangle + |V_aV_b\rangle)/\sqrt{2}$ state.

$|\Phi_{a,b}\rangle$ is then distributed to four nodes using fiber beam splitters (FBS) with a fiber spool of 3 km, respectively, and we can prepare our probe state $|\Psi_4^2\rangle$, which is a superposition of the Bell state between two adjacent nodes. We implemented phase encoding using a combination of two QWPs (with an optic axis angle 45°) and a HWP as shown in Supplementary Figure 1 [1]. After encoding four phases $\boldsymbol{\phi}$, the probe state $|\Psi_4^2\rangle$ evolves into $\hat{U}(\boldsymbol{\phi})|\Psi\rangle$. Then, projective measurements are performed on a σ_x basis, which is realized by a HWP with an optic axis angle

of 22.5° and a PBS. The two-photon coincidence counts are then measured between the two different nodes using superconducting nanowire single-photon detectors (SNSPDs).

B. Two-photon probability results

After the phase encoding $\hat{U}(\boldsymbol{\phi})$, the probe state $|\Psi_4^2\rangle$ evolves as follows:

$$\hat{U}(\boldsymbol{\phi})\,|\Psi_4^2\rangle = \frac{1}{2}\,[(|H_1H_2\rangle + e^{i(\phi_1+\phi_2)}|V_1V_2\rangle) + (|H_2H_3\rangle + e^{i(\phi_2+\phi_3)}|V_2V_3\rangle) + (|H_3H_4\rangle + e^{i(\phi_3+\phi_4)}|V_3V_4\rangle) + (|H_4H_1\rangle + e^{i(\phi_4+\phi_1)}|V_4V_1\rangle)]. \tag{1}$$

In the experiment, we performed a σ_x measurement which can observe the maximum visibility for the interference fringe [2]. After measuring the two-photon coincidence detection using the SNSPDs with a set of positive operator-valued measures (POVM) $\{|+_1+_2\rangle\langle+_1+_2|, |+_1-_2\rangle\langle+_1-_2|, |-_1+_2\rangle\langle-_1+_2|, |-_1-_2\rangle\langle-_1-_2|, |+_2+_3\rangle\langle+_2+_3|, |+_2-_3\rangle\langle+_2-_3|, |-_2+_3\rangle\langle-_2+_3|, |-_2-_3\rangle\langle-_2-_3|, |+_3+_4\rangle\langle+_3+_4|, |+_3-_4\rangle\langle+_3-_4|, |-_3+_4\rangle\langle-_3+_4|, |-_3-_4\rangle\langle-_3-_4|, |+_4+_1\rangle\langle+_4+_1|, |+_4-_1\rangle\langle+_4-_1|, |-_4+_1\rangle\langle-_4+_1|, |-_4-_1\rangle\langle-_4-_1|\}$ where $|+_i\rangle$ ($|-_i\rangle$) is a diagonal (anti-diagonal) polarization basis $|D\rangle = (|H\rangle + |V\rangle)/\sqrt{2}$ ($|A\rangle = (|H\rangle - |V\rangle)/\sqrt{2}$) at node i, we have the following set of theoretical probabilities:

$$\begin{aligned}
P_{12}^{++} = P_{12}^{--} &= \frac{1+\mathrm{V}_{12}^{\pm\pm}\cos(\phi_1+\phi_2)}{16}, \quad P_{12}^{+-} = P_{12}^{-+} = \frac{1-\mathrm{V}_{12}^{\pm\mp}\cos(\phi_1+\phi_2)}{16},\\
P_{23}^{++} = P_{23}^{--} &= \frac{1+\mathrm{V}_{23}^{\pm\pm}\cos(\phi_2+\phi_3)}{16}, \quad P_{23}^{+-} = P_{23}^{-+} = \frac{1-\mathrm{V}_{23}^{\pm\mp}\cos(\phi_2+\phi_3)}{16},\\
P_{34}^{++} = P_{34}^{--} &= \frac{1+\mathrm{V}_{34}^{\pm\pm}\cos(\phi_3+\phi_4)}{16}, \quad P_{34}^{+-} = P_{34}^{-+} = \frac{1-\mathrm{V}_{34}^{\pm\mp}\cos(\phi_3+\phi_4)}{16},\\
P_{41}^{++} = P_{41}^{--} &= \frac{1+\mathrm{V}_{41}^{\pm\pm}\cos(\phi_4+\phi_1)}{16}, \quad P_{41}^{+-} = P_{41}^{-+} = \frac{1-\mathrm{V}_{41}^{\pm\mp}\cos(\phi_4+\phi_1)}{16},
\end{aligned} \tag{2}$$

where $\mathrm{V}_{jk}^{\pm\pm}$ and $\mathrm{V}_{jk}^{\pm\mp}$ are the visibility of two-photon interference fringes and $\{jk\} = \{12, 23, 34, 41\}$. We obtained the experimental outcome probability results by scanning ϕ_1 and ϕ_3 while we fixed ϕ_2 and ϕ_4. The experimental results are shown in Supplementary Figure 2 and the solid lines in Supplementary Figures 2 are drawn based on our theoretical model with interference visibility in Supplementary Equation (2).

The experimentally obtained visibilities of P_{12}^{++}, P_{12}^{+-}, P_{12}^{-+}, P_{12}^{--}, P_{23}^{++}, P_{23}^{+-}, P_{23}^{-+}, P_{23}^{--}, P_{34}^{++}, P_{34}^{+-}, P_{34}^{-+}, P_{34}^{--}, P_{41}^{++}, P_{41}^{+-}, P_{41}^{-+}, and P_{41}^{--} are 0.955, 0.981, 0.970, 0.945, 0.975, 0.977, 0.965, 0.982, 0.953, 0.960, 0.946, 0.948, 0.979, 0.986, 0.982, and 0.978, respectively. Supplementary Figure 3 shows our experimental results on the outcome probabilities by scanning $\boldsymbol{\phi}$ simultaneously. In Supplementary Figure 3, data points are normalized by the measured two-photon coincidence counts around 36,700, which was obtained by summing $\bar{\mu} \simeq 367$ measurements 100 times [3]. Each surface corresponds to a theoretical model with experimentally measured visibility values.

We now estimate an average of unknown phase $\hat{\phi}$ of Figure 4**c** in the main text using the maximum likelihood estimation (MLE), which is maximizing the log-likelihood function consisting of the product of sixteen probabilities [4]:

$$L = \log(P_{12}^{++C_0} \times P_{12}^{+-C_1} \times P_{12}^{-+C_2} \times \cdots \times P_{41}^{--C_{15}}), \tag{3}$$

where C_j is a two-photon coincidence count with $j = \{0, 1, \ldots, 15\}$. Then the estimated phases of $\hat{\phi}_{\text{est}}$ and standard deviation $\Delta\hat{\phi}_{\text{est}}$ can be obtained by maximizing Supplementary Equation (3). Uncertainty of standard deviation was obtained from the approximation of $\delta(\Delta\hat{\phi}) = \Delta\hat{\phi}/\sqrt{2(s-1)}$ [5, 6]. We performed the $\bar{\mu} \simeq 367$ measurements 100 times and obtained standard deviations and uncertainties of the estimated phases as shown in the Supplementary Table 1.

C. Generalization

Our scheme can be extended to a general scenario with N-photon and d unknown phases for estimating an average of distributed phases $\hat{\phi} = \sum_{j=1}^{d} \phi_j/d$. We consider that a superposition of the N-photon GHZ states is distributed

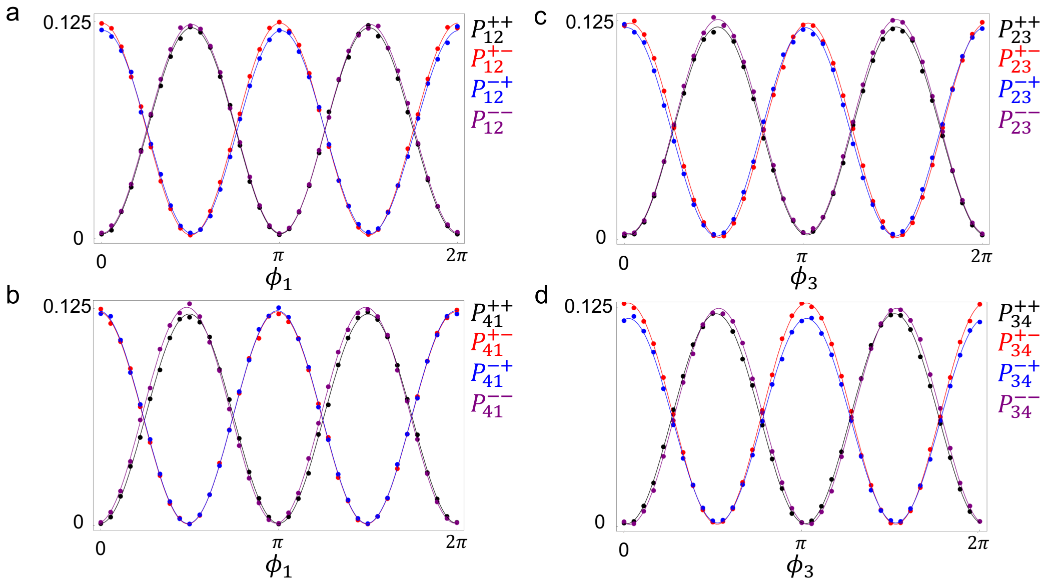

Supplementary Figure 2. **Experimentally obtained outcome probabilities. a-d**, The measured averaged visibilities for the outcome probabilities are 0.970±0.01 with an optical fiber distance of 6 km between the two different nodes. The black, red, blue, and purple lines represent the interference fringe $P_{jk}^{++}, P_{jk}^{+-}, P_{jk}^{-+}$, and P_{jk}^{--} where ϕ_2 and $\phi_4 = 0$.

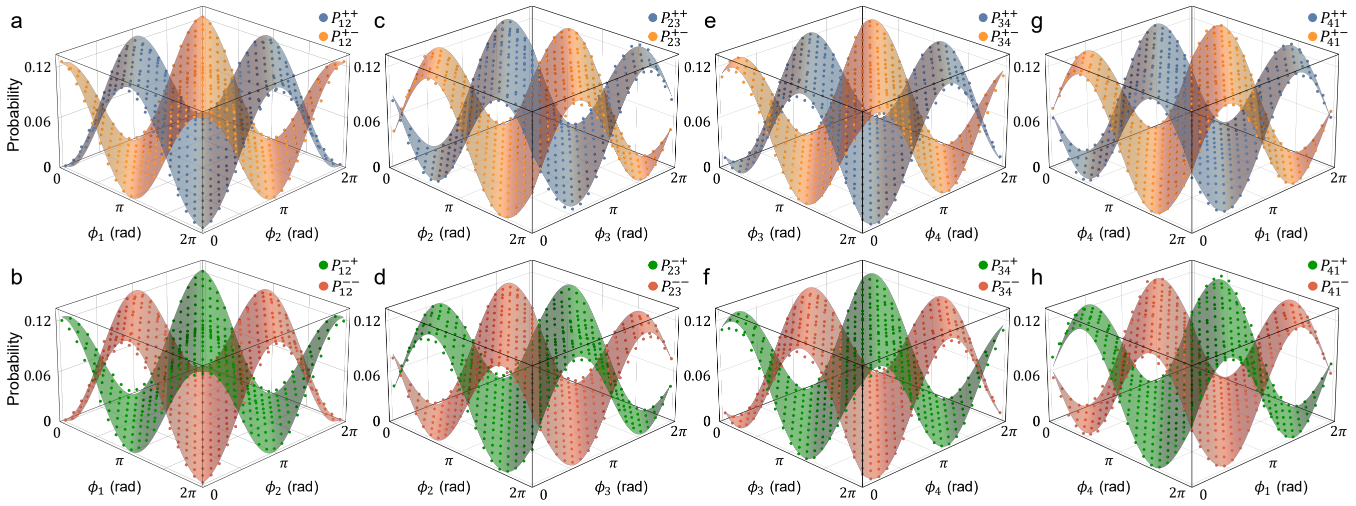

Supplementary Figure 3. **Experimentally obtained $P_{jk}^{\pm\pm}$ by scanning $\boldsymbol{\phi}$. a-h**, Surface plots are drawn from fitting functions of $P_{jk}^{\pm\pm}$ with $\{jk\} = \{12, 23, 34, 41\}$ by scanning ϕ_1, ϕ_2, ϕ_3, and ϕ_4. The blue, orange, green, and red surfaces correspond to P_{jk}^{++}, P_{jk}^{+-}, P_{jk}^{-+}, and P_{jk}^{--}, respectively. All error bars are smaller than the size of the markers.

to the two adjacent nodes out of d nodes. The extended probe state under phase encoding, when N is even, can be described as follows:

$$
\begin{aligned}
|\Psi_d^N\rangle &= \frac{1}{\sqrt{d}}(|\Phi_{1,2}^N\rangle + |\Phi_{2,3}^N\rangle + ... + |\Phi_{d-1,d}^N\rangle + |\Phi_{d,1}^N\rangle) \\
&= \frac{1}{\sqrt{2d}}\sum_{j=1}^{d}\left(|H_j\rangle^{\otimes N/2}|H_{j+1}\rangle^{\otimes N/2} + |V_j\rangle^{\otimes N/2}|V_{j+1}\rangle^{\otimes N/2}\right) \qquad (4)
\end{aligned}
$$

$$
\xrightarrow{\text{Phase Encoding } \boldsymbol{\phi}} \hat{U}(\boldsymbol{\phi})|\Psi_d^N\rangle = \frac{1}{\sqrt{2d}}\sum_{j=1}^{d}\left(|H_j\rangle^{\otimes N/2}|H_{j+1}\rangle^{\otimes N/2} + e^{i\frac{N}{2}(\phi_1+\phi_2)}|V_j\rangle^{\otimes N/2}|V_{j+1}\rangle^{\otimes N/2}\right), \qquad (5)
$$

	$\hat{\phi}_{\rm est}$ (rad)	$\Delta\hat{\phi}_{\rm est}$ ($\times 10^{-2}$)	$\Delta\hat{\phi}_{\rm cal}$ ($\times 10^{-2}$)
1	0.07495	2.799±0.199	2.946
2	0.16201	2.746±0.195	2.851
3	0.25210	2.867±0.204	2.833
4	0.34005	2.729±0.194	2.871
5	0.42353	2.725±0.194	2.996

Supplementary Table 1. Experimentally obtained estimated phase $\hat{\phi}_{\rm est}$ and standard deviation $\Delta\hat{\phi}_{\rm est}$. Standard deviation $\Delta\hat{\phi}_{\rm cal}$ is calculated based on sixteen probabilities with interference visibility.

where $d+1 \equiv 1$, for instance, $|H_{d+1}\rangle \equiv |H_1\rangle$, and the subscript denotes the node, for instance, $|\Phi^N_{1,2}\rangle$ denotes the N-photon GHZ states $|\Phi^N_{1,2}\rangle = (|H_1\rangle^{\otimes N/2}|H_2\rangle^{\otimes N/2} + |V_1\rangle^{\otimes N/2}|V_2\rangle^{\otimes N/2})/\sqrt{2}$ between the node 1 and the node 2, meaning that $N/2$ photons are at the node 1 and $N/2$ photons are at the node 2, respectively. The generalized probe states $|\Psi^N_d\rangle$ can be interpreted as a superposition of d GHZ states between the two adjacent nodes. Projective measurements on GHZ states are conducted on the $\sigma_x^{\otimes N}$ basis. In this condition, it yields a corresponding probability set $\{P^{\pm}_{jk,l}\}$, where j and k denote nodes i.e., $\{jk\} = \{12, 23, \ldots, (d-1)d, d1\}$, and l means the number of probabilities for the superscript + and −, i.e., $\{l\} = \{1, 2, \ldots, 2^{n-1}d\}$. Then, the outcome probability for $|\Psi^N_d\rangle$ can be written as,

$$P^{\pm}_{jk,l} = \frac{1 \pm \cos\frac{N}{2}(\phi_j + \phi_k)}{2^N d}, \tag{6}$$

For example, when $N = 2$ and $d = 4$, the outcome probability set corresponds to Supplementary Equation (2). Using Supplementary Equation (6), the corresponding Fisher information matrix can be calculated as follows:

$$\mathbf{F} = \begin{pmatrix} N^2/2d & N^2/4d & 0 & \cdots & 0 & N^2/4d \\ N^2/4d & N^2/2d & N^2/4d & 0 & \cdots & 0 \\ 0 & N^2/4d & N^2/2d & N^2/4d & \cdots & \vdots \\ \vdots & 0 & N^2/4d & \ddots & \cdots & 0 \\ 0 & \vdots & \vdots & \vdots & \ddots & N^2/4d \\ N^2/4d & 0 & 0 & \cdots & N^2/4d & N^2/2d \end{pmatrix}. \tag{7}$$

Then, from Supplementary Equation (7), the weak form of Cramer-Rao bound for an average of distributed phase $\hat{\phi} = (\phi_1 + \phi_2 + \cdots + \phi_d)/d$ is calculated to be $\Delta^2\hat{\phi} = 1/N^2$, which corresponds to the Heisenberg scaling.

D. Analysis with experimental imperfections

We derive the losses of experimental setup as shown in Figures **2a-b** in the main text. First, we analysis the sensitivity for our performed experiment with considering losses: 1) collection efficiency of the Bell state $\eta_{\rm b}$, 2) optical transmission at a fiber beam splitter $\eta_{\rm bs}$, 3) fiber spool $\eta_{\rm fs}$, 4) collection efficiency before the SNSPD $\eta_{\rm c}$, 5) detection efficiency of SNSPD $\eta_{\rm d}$, and 6) other losses from optical components $\eta_{\rm etc}$. Our propose state considering loss becomes $|\psi^2_4\rangle = \eta_{\rm tot}|\psi^2_4\rangle$, where $\eta_{\rm tot} = \eta_{\rm b}\eta_{\rm bs}\eta_{\rm fs}\eta_{\rm c}\eta_{\rm d}\eta_{\rm etc}$. After the phase encoding and conduct by projective measurement in σ_x basis at each node, we can obtain sixteen probabilities as follows:

$$\begin{aligned} P^{++}_{jk} = P^{--}_{jk} = \eta^2_{\rm tot}\frac{(1 + {\rm V}\cos(\phi_j + \phi_k))}{16}, \\ P^{+-}_{jk} = P^{-+}_{jk} = \eta^2_{\rm tot}\frac{(1 - {\rm V}\cos(\phi_j + \phi_k))}{16}, \end{aligned} \tag{8}$$

where V is the visibility of $P^{\pm\pm}_{jk}(P^{\pm\mp}_{jk})$ and $\{jk\} = \{12, 23, 34, 41\}$. Then, using Supplementary Equation (8), the Fisher information can be calculated as $4\eta^2_{\rm tot}{\rm V}^2$ and corresponding $\Delta\hat{\phi}$ is calculated to be $1/\sqrt{4\eta^2_{\rm tot}{\rm V}^2}$. Then, the

threshold for violation of the standard quantum limit (SQL) is $\Delta\hat{\phi} \leq \Delta\hat{\phi}_{\mathrm{SQL}} = 1/\sqrt{2}$. In our experiments, averaged total transmission $\eta_{\mathrm{tot}}^2 = 0.097$ and $\mathrm{V}_{\mathrm{ave}} = 0.967$ are obtained as shown in Supplementary Table 2, and corresponding $\Delta\hat{\phi}$ can be calculated as 1.63 without post-selection. Therefore, to violate the SQL, total efficiency is required $\eta_{\mathrm{tot}} \geq 1/\sqrt{2}\mathrm{V}_{\mathrm{ave}} \approx 0.731$. It is now clear that unconditional violation with our proposed states can be achieved with state-of-the-art technologies such as high-efficiency SNSPD, optimized alignment, and low-loss optical components.

Efficiency	Ours	State-of-the-art
η_{b}	0.78	0.95 [7]
η_{bs}	0.95	0.95
η_{fs}	0.83	0.88
η_{c}	0.66	0.95 [7]
η_{d}	0.80	0.98 [8]
η_{etc}	0.96	1
η_{etc}^2	0.097	0.55

Supplementary Table 2. Experimentally obtained efficiencies of our setup and the state-of-the-art efficiencies.

Supplementary References

[1] S. Hong, Y.-S. Kim, Y.-W. Cho, S.-W. Lee, H. Jung, S. Moon, S.-W. Han, H.-T. Lim, et al., Quantum enhanced multiple-phase estimation with multi-mode $N00N$ states, Nature Communications **12**, 1 (2021).

[2] K. J. Resch, K. L. Pregnell, R. Prevedel, A. Gilchrist, G. J. Pryde, J. L. O'Brien, and A. G. White, Time-reversal and super-resolving phase measurements, Physical Review Letters **98**, 223601 (2007).

[3] L. Pezze, and A. Smerzi, Quantum theory of phase estimation, Preprint at https://arxiv.org/abs/1411.5164 (2014).

[4] E. Polino, M. Valeri, N. Spagnolo, and F. Sciarrino, Photonic quantum metrology, AVS Quantum Science **2**, 024703 (2020).

[5] L.-Z. Liu, Y.-Z. Zhang, Z.-D. Li, R. Zhang, X.-F. Yin, Y.-Y. Fei, L. Li, N.-L. Liu, F. Xu, Y.-A. Chen, et al., Distributed quantum phase estimation with entangled photons, Nature Photonics **15**, 137 (2021).

[6] S.-R. Zhao, Y.-Z. Zhang, W.-Z. Liu, J.-Y. Guan, W. Zhang, C.-L. Li, B. Bai, M.-H. Li, Y. Liu, L. You, et al., Field demonstration of distributed quantum sensing without post-selection, Physical Review X **11**, 031009 (2021).

[7] Weston, M. M. et al. Efficient and pure femtosecond-pulse-length source of polarization-entangled photons. Opt. Express **24**, 10869-10879 (2016).

[8] Reddy, D. V., Nerem, R. R., Nam, S. W., Mirin, R. P. & Verma, V. B. Superconducting nanowire single-photon detectors with 98% system detection efficiency at 1550 nm. Optica **7**, 1649–1653 (2020).
